# Classical and Recent Developments of Membrane Processes for Desalination and Natural Water Treatment

**DOI:** 10.3390/membranes12030267

**Published:** 2022-02-25

**Authors:** Catherine Charcosset

**Affiliations:** Laboratoire d’Automatique, de Génie des Procédés et de Génie Pharmaceutique (LAGEPP), Université Claude Bernard Lyon 1, 43 bd du 11 Novembre 1918, Bâtiment CPE, 69622 Villeurbanne, France; catherine.charcosset@univ-lyon1.fr; Tel.: +33-4-72-43-18-34

**Keywords:** water, membrane, membrane process, treatment, desalination, contaminants

## Abstract

Water supply and water treatment are of major concern all around the world. In this respect, membrane processes are increasingly used and reported for a large range of applications. Desalination processes by membranes are well-established technologies with many desalination plants implemented in coastal areas. Natural water treatment is also well implemented to provide purified water for growing population. This review covers various aspects of desalination: membranes and modules, plants, fouling (scaling, biofouling, algal blooms), cleaning, pretreatment (conventional and membrane treatments), energy and environmental issues, renewable energies, boron removal and brine disposal. Treatment of natural water focuses on removal of natural organic matter, arsenic, iron, nitrate, fluoride, pesticides and herbicides, pharmaceutical and personal care products. This review underlines that desalination and natural water treatment require identical knowledge of membrane fouling, construction of large plants, cleaning procedures, energy and environmental issues, and that these two different fields can learn from each other.

## 1. Introduction

Due to the increasing need for fresh water both for human consumption and plant irrigation, desalination and treatment of natural water are being increasingly developed. For seawater and brackish water desalination, reverse osmosis (RO) has become a major process. Other membrane techniques used for desalination include forward osmosis (FO), membrane distillation (MD) and electrodialysis (ED). Treatment of natural water (ground and surface water) is also a major activity in several places in the world. As natural water may contain several contaminants (natural organic matter (NOM), iron, and fluoride, among others), their removal is required.

This review covers various aspects of desalination, including membranes and modules, typical plants, fouling (scaling, biofouling, algal blooms), cleaning, pretreatment before RO (conventional and membrane treatments), energy and environmental issues, renewable energies for desalination, boron removal and brine disposal. Treatment of natural water is presented, including removal of NOM, arsenic, iron, nitrate, fluoride, pesticides and herbicides, pharmaceuticals and personal care products.

## 2. Previous Reviews

Due to the huge development of desalination over the past 40 years, several reviews have been written. Some of these reviews concern the main aspects of desalination. For example, Fritzmann et al. [1] provided a very complete review, including implementation (membranes, modules, membrane cleaning, energy recovery systems), raw water characterization (chemical foulants, particulate fouling, biofouling, organic foulants), pretreatment (chemical, conventional, membrane), post-treatment (recarbonation and remineralisation, disinfection, boron removal), waste management and environmental impact, energy requirement and cost. Greenlee et al. [2] summarized the history of desalination, composition of feed waters (sea water and brackish water), membrane fouling, membrane cleaning, RO system design, pretreatment and post-treatment, RO concentrate disposal, alternative energy sources, and costs.

RO membrane materials for desalination have been the subject of specific review papers [3,4]. Lee et al. [3] focused on RO membrane materials for desalination, their development and future potential. Conventional desalination RO membranes include thin film composite membranes, membrane post-synthesis modifications and control of interfacial polycondensation reactions. Among novel desalination RO membranes are polymeric membranes with rigid star amphiphiles, ceramic/inorganic membranes and mixed matrix membranes (nanoparticle/polymeric membranes, carbon nano-tube/polymeric membranes). Shenvi et al. [5] focused their review on RO membrane materials and modules, as well as problems associated with RO modules such as scaling, boron removal and brine disposal. Qasim et al. [6] discussed theories and models related to concentration polarization and membrane transport, membrane modules, membrane cleaning and different pretreatment technologies, membrane fouling, process design, economic and energy considerations, as well as current challenges faced by RO desalination processes.

Some reviews are devoted to specific aspects of seawater RO desalination. For example, Miller et al. [7] focuses on environmental issues associated to desalination and solutions proposed. Energy required for desalination is one of the most important environmental problems. Elimelech and Phillip [8], Kim et al. [9], Park et al. [10], Nassrullah et al. [11] reviewed possible reductions in energy demand, focusing on advances in materials, and innovative technologies in improving RO performance. Matin et al. [12,13] focused on biofouling and scaling in RO membranes during seawater desalination, Villacorte et al. [14] on seawater desalination and harmful algal blooms, while Ghaffour et al. [15], Kalogirou et al. [16], Charcosset [17], Bundschuh et al. [18] reviewed renewable energies for desalination. Other reviews focus on alternative techniques to RO, such as FO, MD and ED [19,20,21,22,23].

Several reviews are available on the treatment of natural waters for the removal of specific pollutants, including boron [24], iron [25], nitrate [26], fluoride [27,28], pesticides [29], and pharmaceutical and personal care products [30,31].

The aim of the present article is to provide a state-of-the-art study on water treatment by membrane processes by focusing both on seawater (desalination) and natural water treatment. Both seawater and natural water contain low amounts of toxic compounds (much less that wastewater). They are treated by similar processes (RO, NF, UF, MF.), raise similar questions (productivity and cost, environmental issues, membrane fouling) and need both the development of similar techniques and new understanding of related phenomena. The review has two main sections. Section 3 is related to desalination, and presents membranes and modules used in RO, operation of typical RO desalination plants, pretreatment prior to RO, other membrane processes for desalination, topics related to energy, environmental issues, boron removal and renewable energies for desalination. The treatment of natural water is presented in Section 4, focusing on the removal of natural organic matter, arsenic, iron, nitrate, fluor, pesticides and herbicides and finally pharmaceuticals and personal care products. The review is based on previous reviews and new articles to give a general background in the field of seawater and natural water treatments, as well a description of some new findings in this area.

## 3. RO Desalination

### 3.1. Introduction

Nowadays, water scarcity is one of the most serious global challenges [8]. The need for fresh water is a critical problem, as climate change. According to the World Health Organization (WHO), there are more than 2.5 billion people (about 40% of the world’s population) who do not have access to drinking water. As a consequence of the growing scarcity of fresh water, the implementation of large desalination plants has been increasing over the past years. Generally, desalination processes are categorized into two major types: (1) phase-change/thermal and (2) membrane processes. Some of the phase-change processes include compression, freezing, humidification/dehumidification and solar stills. Membrane based processes include RO, MD and ED. RO technology has improved considerably in the past two decades, and current desalination plants can desalinate seawater with much less energy than thermal desalination. Currently, the largest seawater RO plant in the world is in Ashkelon (Israel), with a production rate of about 110 million m^3^/year [8]. RO is driven by transmembrane pressure during ultrafiltration (UF) and microfiltration (MF); however, in RO, the water flux through the membrane is proportional to the applied pressure minus the osmotic pressure of the solution on the membrane side opposite to the feed solution. Desalination is a major application of RO.

### 3.2. Membranes and Modules 

Membranes and modules have been intensively studied [1,3,6]. RO membranes are usually asymmetric. The support layer protects the membrane from breaking, while the active layer provides selectivity of the membrane. In the early 1960s, the first asymmetric RO membranes were prepared by Loeb and Sourirajan [32]. Later, in the early 1970s, the first commercially available RO membranes made of cellulose acetate were introduced into the market. One of the major drawbacks of cellulose acetate membranes is the possibility of membrane deterioration by hydrolysis. In addition, cellulose acetate membranes tend to strongly compact under high pressure and flux and overall performance decreases. Although cellulose acetate membranes are still commercially available, thin-film composite membranes are mostly used.

**Figure 1:**
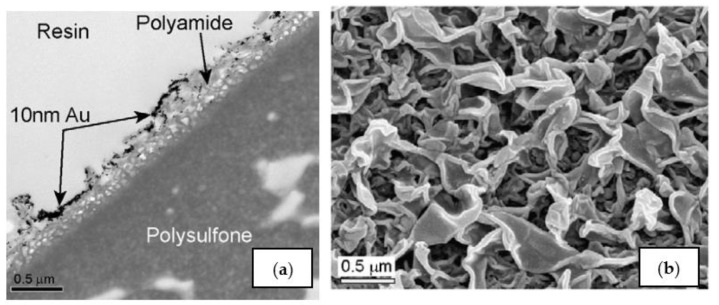
TEM cross-section of a typical RO membrane (ESPA3, Hydranautics) previously used in a filtration experiment with 10 nm gold nanoparticles [33]. (**a**)TEM of the membrane cross-section. Gold nanoparticles are used to obtain sufficient contrast for imaging. (**b**) SEM of the polyamide top surface showing the typical rough ridge and valley structure from [33].

In a thin-film composite membrane, the supporting layer is usually a UF or MF membrane made of polysulphone and the active layer from polyamide (Figure 1). These membranes have several advantages, such as chemical and mechanical stability, and resistance to bacteria degradation, and are less influenced by membrane compaction. However, composite membranes are less hydrophilic and therefore have a stronger tendency for fouling than cellulose acetate membranes. During these last 20 years, membrane performance has significantly increased with respect to both permeability and salt rejection. The rejection of typical seawater membranes is 99.8%, while flux is around 69 L/(m^2^ day bar).

RO polyamide membranes can be produced using monomeric aromatic amines and aromatic acyl halides containing at least three carbonyl halide groups, such as trimesoyl chloride. The four major membrane module suppliers (DOW, Toray, Hydranautics and Toyobo) provide such RO membranes for large scale desalination plants. The FT-30 membrane (DOW FILMTEC^TM^) is produced by reaction of 1,3-benzenediamine and trimesoyl chloride. Its morphology is unique, as its pores have a “ridge and valley” shape. A number of similar membranes are available, e.g., the CPA2 membrane produced by Hydranautics and the UTC-70 by Toray Industries.

Despite great improvements in thin-film composite membranes, they still suffer from several limitations [8]. Hollow fiber configurations that offer higher packing densities have not been successfully produced. In addition, the surface properties of thin-film composite membranes make them prone to fouling, which diminishes process performance.

Spiral wound membrane devices are most used for RO desalination. They have the advantages of a high specific membrane surface area, easy scaling up, easy changeability, and low replacement cost. In addition, the spiral wound module is the least expensive to produce from a flat sheet composite membrane. The current industrial standard element measures 8-in. in diameter. Inside one pressure vessel, four to eight elements are placed in series with a connected permeate collector tube to minimise piping and reduce the number of pipe connections. Larger modular elements are available for increased desalination capacity. Koch Membrane Systems introduced the large diameter (MegaMagnum^TM^) element with a nominal diameter of 18-inches, and 16-inch modules are commercialized by Hydranautics and DOW (FILMTEC^TM^).

New membranes for RO can be obtained by modifying the membrane surface properties. For many years, various chemical and physical techniques have been developed, such as coating the membrane surface with more hydrophilic compounds, and chemical treatments. Other surface modification techniques include the use of free radical-, photochemical-, radiation-, redox- and plasma-induced reaction. A key point on membrane science for desalination is to develop antifouling RO membranes. Zhao et al. [34] summarized the three main strategies to obtain fouling resistant thin-film composite RO membranes: substrate modification before interfacial polymerization, incorporating (hydrophilic/biocidal/antifouling) molecules into the thin layer during interfacial polymerization, and post (surface) modification after interfacial polymerization. Many researches in this field are still going on, with promising results in terms of membranes with anti-scaling and anti-fouling properties, although availability, large-scale use and long-term stability in RO plants need to be confirmed [35,36]. Another direction in membrane development for RO is the development of chlorine-resistant RO membranes, to eliminate the need for neutralization and improve resistance to biofouling [37].

New materials for RO have emerged as the consequence of nano-technology science development. These novel membranes include zeolite membranes, thin film nano-composite membranes, carbon nano-tube membranes, and aquaporin-based membranes. These have advantages compared to traditional RO composite membranes such as better selectivity, but their availability and large-scale application remain challenging [3,8,38]. In addition, these materials have a relatively low impact on increasing energy efficiency [4]. Research efforts are still needed to optimize water-salt selectivity rather than membrane permeability to enhance desalination process efficiency.

### 3.3. Operation

**Figure 2:**
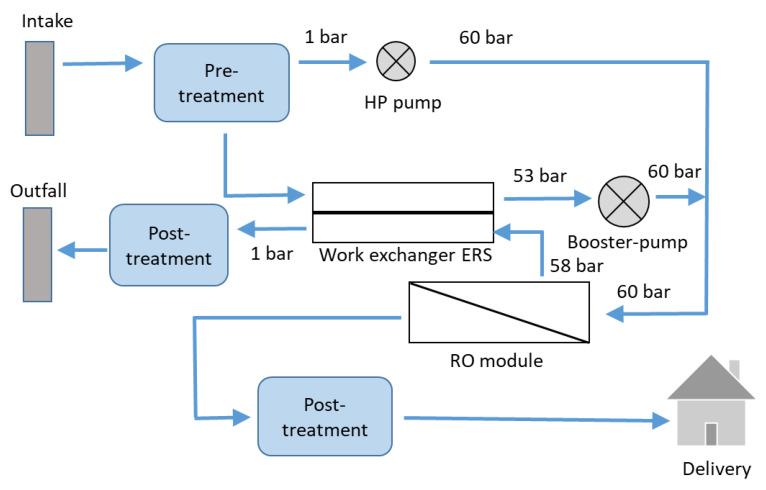
Simplified reverse osmosis scheme with an energy recovery system (adapted from [1]).

A typical RO desalination plant includes RO modules with an energy recovery system and open seawater intake. The process includes the following stages (Figure 2).

- The abstraction of feed water can be realised either through coastal and beach wells or through open seawater intake systems [1]. Abstraction through wells has several advantages: the water quality is better, with less turbidity, and less algae and total dissolved solids. However, wells require more space. In brackish water desalination, the abstraction of feed water is realized through wells.
- In the pretreatment stage, colloids are removed from the feed water and chemicals are added to prevent scaling and fouling. The composition and pH of the intake water are adjusted. Pretreatment has a major influence on the RO performance by lowering the fouling propensity of the RO membranes. A specific section of this review is dedicated to pretreatment.
- A pump is used to create the required transmembrane pressure and to overcome the height differences within pipes in the RO plant. High transmembrane pressure must be used (up to 7000 kPa) due to the high salt concentrations of seawater. The power required to pump the feed water is directly related to the feed pressure and flow rate [2].
- The RO membranes separate salt from water with a rejection of 98–99.5%, depending on the membranes used. Several RO plants operate with either one, two or four RO passes [2]. The choice between one or more RO passes depends on several factors, including energy cost, feed water, desired recovery, and product water standards.
- The energy recovery system is aimed at transferring the potential energy from the concentrate to the feed. Current energy recovery systems operate with efficiencies greater than 95%. Several energy recovery devices are available. The most common uses hydraulic power to cause a positive displacement within the recovery device [2]. Several RO plants use these devices, such as the DWEER (DWEER Technology, Ltd., George Town, Cayman Islands), PX Exchanger (ERI), or PES (Siemag’s Pressure Exchanger System) [2].

In the post-treatment step, permeate is re-mineralised, re-hardened, disinfected by chlorination and adjusted to drinking water standards [1]. Alkalinity is added to water to make it nonaggressive and noncorrosive. This is done by dissolution of lime or limestone by carbon dioxide or addition of a calcium chloride or bicarbonate solution. Disinfection of produced RO water is done by adding chlorine, hypochlorite or sodium hypochlorite. In addition, post-treatment methods must be applied for boron removal to avoid toxic effects of boron on humans and agriculture. Under standard test conditions, seawater RO high rejection membranes display boron rejection between 88% and 91%. BWRO membranes reject between 30% and 80% of the uncharged boron compound. In general, ion exchange resins are used to remove boron from RO water. Typically, boron removal using ion exchange resins is performed in one step, with removal higher than 99–99.99%.

In addition, a control system is necessary to maintain continuous and reliable production.

**Figure 3:**
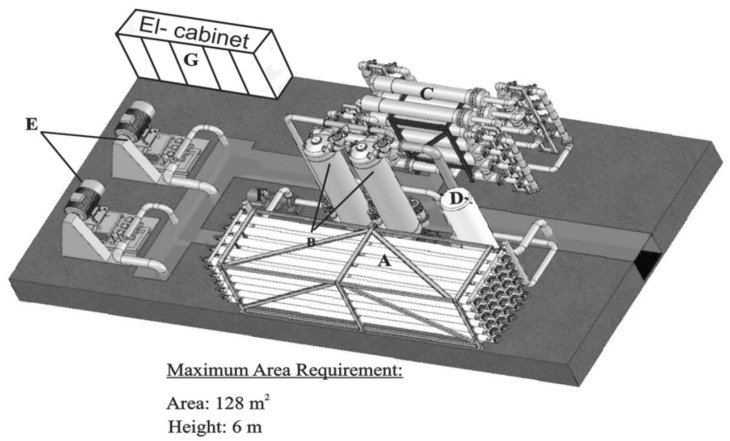
Typical RO stage installation. (A) Pressure vessel, membranes and manifolds; (B) pressure recuperator towers; (C) bag filters; (D) seawater feeding tank; (E) high pressure pumps; (F) booster pump; (G) electro-cabinet (from [1]).

A typical RO stage installation is shown in Figure 3.

### 3.4. Boron Removal

Boron is known to be toxic to both humans and plants, even at low concentrations. Although the WHO has increased the recommended maximum concentration (from 0.3, to 0.5 and 2.4 mg/L in 2011), boron removal by RO remains a difficult task as boron is present in seawater in its uncharged boric acid form, which can pass through the RO membrane. The increase in pH above the boric acid pKa (9.2 at 25 °C) converts boric acid into negatively charged borate anions, enhancing boron rejection by RO membranes [39]. Therefore, RO desalination plants can be implemented in a double-pass configuration where the pH of the RO permeate obtained from the first pass is chemically increased above the pKa of H_3_BO_3_, and is treated again using RO as a second pass. High boron removal efficiency is then obtained. However, the technique involves higher operating and capital costs [40].

Several other techniques are available for boron removal, such as ion exchange, chemical precipitation, adsorption, and electrocoagulation [24]. In RO desalination plants, the most popular technique uses ion exchange resins specifically designed for boron removal. These commercial resins (e.g., Amberlite IRA743, Purolite S108, Diaion CR05) have a macroporous polystyrene matrix on which N-methyl-D-glucamine functional groups are attached. The ion exchange technique is highly efficient for boron removal but its disadvantages include the use of costly chemicals for regeneration and their disposal [5].

Ion exchange resins can also be used in a hybrid process in which a reactor is associated with an UF or MF membrane (submerged or not) [41]. In most configurations, boron solution and fresh resin are continuously added to the reactor, while saturated resins are removed at the same flowrate by MF [41]. The technique can also be performed without continuous addition of resin [42]. Ion exchange resins of small size are used to increase the kinetics of sorption; consequently, boron is retained before passing in the permeate. The major advantages of the technique are that the kinetics and process efficiency are increased.

### 3.5. Fouling

**Figure 4:**
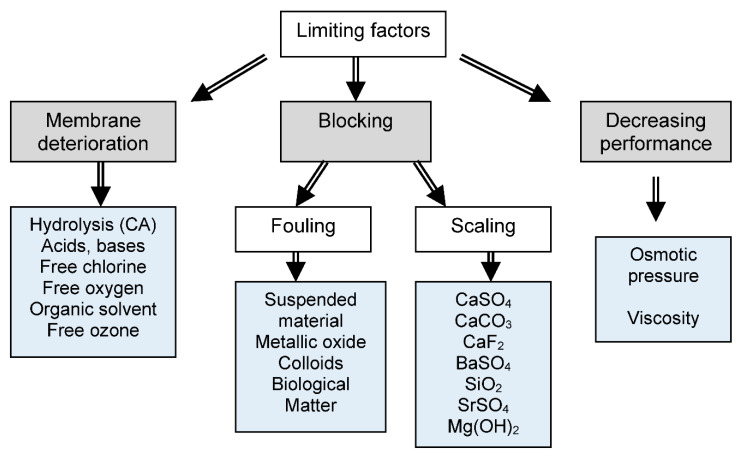
Limiting factors to membrane desalination by reverse osmosis (adapted from [1]).

In RO membranes, fouling types are classified as inorganic salt precipitation (scaling), organic, colloidal, and microbiological (usually bacterial biofilm formation) [43] (Figure 4). Fouling is a major phenomenon in RO desalination that results in a significant increase in operation and maintenance costs. Fouling includes scaling by salts, biofouling by microorganisms and nutricients, and algal blooms.

#### 3.5.1. Scaling

During RO, the concentration of different soluble salts increases in the RO feed channel. When the solubility limit of these salts reaches supersaturation, they precipitate and build a thin layer (scale) on the membrane surface that affects the performance of the RO process, decreasing permeate flux and membrane longevity, leading to higher operating costs [5]. These salts include silica, iron, barium sulfate, calcium carbonate, and gypsum. They are present at different concentrations depending on the source of the feed water. For some compounds, scaling can be removed by flushing the membrane with acid; however, it is often not possible to transport the crystalline mud out of the modules, especially in spiral wound modules.

Therefore, several pretreatments have been proposed to prevent scaling. Anti-scaling agents are widely used as they increase the threshold for the onset of scale formation on the membrane surface. They affect the kinetics of mechanisms involved in crystallization: nucleation and growth. Commonly used anti-scalants include organic polymers, surface active reagents, organic phosphonates and phosphates. Commercial anti-scalants include, for example, Permatreat 510 (a blend of polymers and phosphonates), Hypersperse SI 300 UL (multi-polymer), Acumer 5000 (multi-polymer) and Aquafeed EX-105 (an anionic polyelectrolyte) [44]. The inhibition limits are at SiO_2_ concentrations in the range of 240 to 300 mg/L. Dosages recommended by the commercial literature range from 3 to 15 ppm.

Silica scaling is difficult and costly to remove. In particular, removal of silica by use of anti-scalants is difficult due to the varying parameters influencing silica precipitation [5,45]. Carbonate scaling can be avoided as the pH of the feed water is set between 4 and 6. Gypsum scaling can be reduced in inland brackish water feeds by increasing levels of bicarbonate. Gypsum scaling can be reduced by adding bicarbonate. Mineral scaling can also be limited by pretreatment of the feed by pH adjustment, ion exchange, or nanofiltration (NF)/UF, or by a flow reversal mechanism [46]. In order to limit mineral scaling in RO, it has to be detected as early as possible. Several methods have been developed to monitor flux decline (at constant transmembrane pressure) or transmembrane pressure (at constant water flux) in order to determine the early start of mineral scaling [45]. Such approaches include ultrasonic time-domain reflectometry and electrical impedance spectroscopy. Moreover, direct visual membrane surface monitoring and real-time image analysis can provide detection of the onset of mineral scaling, as well as scaling kinetics and scale morphology.

#### 3.5.2. Biofouling

Biofilm growth on the RO membrane surface is due the presence of microorganisms and nutrients in water, and the convective permeate flow through the membrane [43,47]. Biofilm development is a complex event which is influenced by a number of factors including bacteria and membrane properties and operational parameters. The first step in biofilm formation is the transport and attachment of suspended bacterial cells to the solid–liquid interface. Several factors govern the interactions between bacteria and surfaces. Some are linked to bacterial characteristics such as their hydrophobicity, their fimbriae appendages, their flagellar motility, and lipopolysaccharides and extracellular polymeric materials present at their surface. Other factors concern the surface properties (hydrophobicity and rugosity), the hydrodynamics at the membrane surface and the RO device, and water properties (pH, presence of multivalent cations and nutricients, and ionic strength).

Biofouling of RO membranes is always followed by a decrease in permeate water flux, and a decrease in salt rejection may also be observed. Many seawater desalination facilities have been affected by membrane biofouling, such as large desalination plants in Bahrain and in the US Virgin Islands [12]. Different strategies may be employed to minimize the effect of biofouling, such as feed pretreatment, membrane treatment and membrane modification. Feed pretreatment, such as UF or MF, and biocide application, are aimed at eliminating or minimizing the microbial concentration in the feed stream. Membrane surface modification is aimed at preventing the adhesion of bacteria or inactivation of them if adsorbed. For example, silver and titania nanoparticles can be added to the membranes to limit biofouling as they have antibacterial activity.

#### 3.5.3. Algal Blooms 

Algal blooms are defined by a rapid increase in the population of algae in water [14]. They are frequently referred to as “red tides” due to their vibrant color. Algal blooms are mainly due to natural phenomena, but also to human activities that may increase their frequency and importance. Every costal country can be affected. Several algal species may be involved, with different cell sizes, cell densities and effects. Some algal blooms may be harmful, as they may produce neurotoxins that are toxic substances for human and animals. They can lead to fish die-offs, cities cutting off water to residents, or states having to close fisheries. Moreover, they can proliferate in very dense concentrations. Algal blooms produce algal organic matter of different types and concentrations that may be exuded by living algal cells and/or released through lyses of dead cells. Transparent exopolymeric particles are a major component of algal organic matter, are very sticky and a major initiator and/or promoter of biofilm in marine aquatic environments.

The adverse effects in seawater RO plants due to algal blooms are particle/organic fouling of pretreatment systems and biological fouling of RO membranes, mainly due to accumulation of algal organic matter. Toxins that may be produced by algal cells are also a potential issue but only at very low concentrations. The main effects of algal blooms on seawater RO were highlighted between 2008 to 2009 in the Arabian Gulf region during catastrophic algal blooms [48]. Several seawater RO plants had to reduce or stop operations due to fouling of pretreatment systems and/or to unacceptable feed water quality.

Several pretreatments are possible, but the best solution is early detection of the algal bloom. Thus, the systematic measurement of indicators (e.g., algae and transparent exopolymeric particles) is highly recommended. When possible, subsurface intake is a robust pretreatment to protect seawater RO plants from the impact of algal blooms. Intake includes wells (vertical, angle, and radial type) and galleries. Subsurface intakes provide pretreatment (filtration on granular media) of the inlet seawater. A UF membrane with in-line coagulation is also a very efficient treatment capable of maintaining stable operation and feed water quality, even during severe algal blooms.

Fouling of RO membranes during desalination has been intensively investigated. Future directions include the need for fouling measurement under realistic conditions, studying the interactions between membranes, spacer surface and foulants, and the development of environmentally benign antiscalants [13].

#### 3.5.4. Cleaning

In RO desalination, membrane fouling occurs due to mineral scaling, colloidal particles or biofoulants [1]. Fouling is associated with an increased feed channel pressure drop, decreased permeate flux and reduced salt rejection. To restore membrane performance and to avoid membrane damage, membrane cleaning is necessary. In RO desalination, the main cleaning methods are basically the same as in other membrane processes. They include physical and chemical methods [49]. The cleaning efficiency can be evaluated by flux recovery resistance and salt removal.

For chemical cleaning, a variety of chemical agents are commonly used to clean RO membranes. The selection of the cleaning chemical agents depends on the foulant components, their chemical properties and economic factors. Chemical agents can react with the foulants to reduce the cohesion forces between foulants and adhesion of foulants to the membrane surface, making them easily removable. The chemical agents commonly used include acids, bases, surfactants and chelating agents. Acids, such as hydrochloric acid, nitric acid and sulfuric acid, can remove membrane scaling. Basic solutions can remove an organic fouling layer by hydrolysis and solubilization [43]. Sequential cleaning protocols with different chemicals can be used to recover membrane performance [50].

Ethylene diamine tetra acetic acid (EDTA) is a commonly used chelating agent that is very sensitive to solution pH. Surfactants solubilize macromolecules by forming micelles around them to facilitate the removal of the foulants from the membrane surface. Sodium dodecyl sulfate (SDS) is a common surfactant used in membrane cleaning. SDS can adhere to organic matter due to its hydrophobic part, while the hydrophilic head tends to move towards water. SDS can also remove colloidal fouling under proper cleaning conditions. Cleaning efficiency also depends on operational conditions such as cleaning time, crossflow velocity, cleaning solution temperature, and permeation rate.

Physical cleaning is mainly performed by rinsing the RO device with water under high shear conditions. Chemical and physical cleaning can be performed at the same time. Chemical cleaning solubilizes the foulant layer while physical cleaning flows the foulants away from the membrane surface. Other physical cleaning methods have been proposed without stopping the RO plant, such as a backwash cleaning technique that consists of intermittent injection of a high salinity solution through the membrane [51].

### 3.6. Pretreatment Prior to RO

#### 3.6.1. Conventional Treatments

Pretreatment of the feed water is aimed at reducing fouling potential, increasing RO membrane life, maintaining performance level and minimizing scaling on the membrane surface [1]. To characterize the fouling potential of the RO feed water, the Silt Density Index (SDI) parameter is generally used. SDI_15_ values are recommended to be below 3 to minimize fouling.

**Figure 5:**
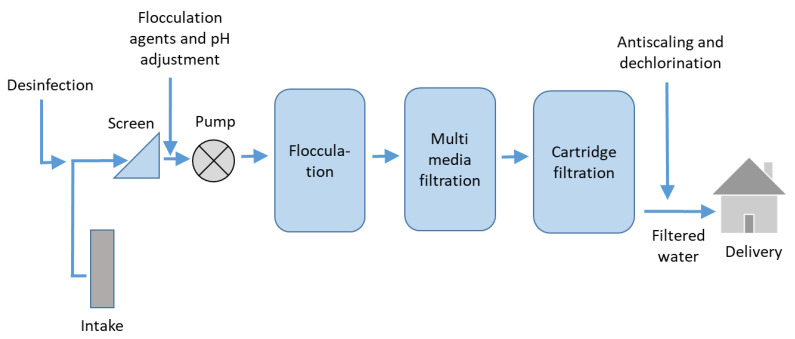
Simplified process scheme of conventional pretreatment of the feed water process before RO desalination (adapted from [1]).

Conventional pretreatments are chemical and physical techniques without the use of membrane processes. A simplified pretreatment process scheme is shown in Figure 5. The chemical pretreatment usually includes coarse pre-filtration on screens, chlorination, acid addition, coagulation, flocculants, sand filtration, addition of antiscalants and sodium bisulphite (to remove residual chlorine) and cartridge filtration (5–10 μm). The physical pretreatment usually consists of flocculation and multimedia filtration followed by cartridge filtration.

Chlorination is aimed at disinfecting the water and preventing biological growth that causes biofouling of filters and RO membranes. For this purpose, chlorine (sodium hypochlorite or chlorine gas), is added to the seawater, where chlorine is hydrolysed to hypochlorous acid.

Addition of an acid, such as sulphuric acid, is aimed at achieving lower pH values at which RO membranes show better performance. To limit CaCO_3_ scaling, the pH is adjusted.

In a conventional pretreatment, coagulation and flocculation agents are often added to cause dissolved matter to adsorb on hydroxides. Sedimentation and sand filtration are then used to remove these agglomerates. To increase the agglomerate size, addition of chemicals is necessary. Coagulants used are ferric chloride FeCl_3_, ferric salts Fe_2_(SO_4_)_3_ or aluminum sulphate Al_2_(SO_4_)_3_, sometimes in combination with polymers.

Antiscalant agents reduce scaling, that is the precipitation of salts (sulphates, carbonates, calcium fluoride, etc) on the membrane surface. Depending upon the salt, different scale inhibitors are used. Sulphuric acid is added to avoid calcium carbonate scaling. Polymeric compounds are commonly used as antiscalants.

Dechlorination of feed water is done before the RO operation because chlorine residue may damage the RO membrane by oxidation. Sodium metabisulphite is commonly used for dechlorination. Activated carbon is also very effective in reducing residual free chlorine.

#### 3.6.2. Membrane Processes

Pretreatment of seawater by UF or MF has been proposed for almost 30 years as an interesting alternative to chemical pretreatment [1]. Suspended particles, colloidal materials, microalgae, bacteria, viruses and pathogenicmicro-organisms are removed from seawater, limiting RO membrane fouling. In addition, UF or MF pretreatments require less chemical addition and space in the RO plant than conventional pretreatments. They are less sensitive to fluctuations in feed water quality and supply the RO stage with superior water quality for long-term operation. They are able to increase the life of RO membrane and can thus lead to overall cost reductions. The efficacy of MF pretreatments was confirmed in a pilot study conducted at Jeddah Port on the Red Sea (Saudi Arabia). Pearce et al. [52] added a UF pretreatment to RO desalination during a 6-month period of algal bloom and storms. The water quality obtained was much better than that observed with the conventional pretreatment.

The use of NF as a pretreatment before RO can reduce water hardness [53]. Indeed, NF membranes are negatively charged and reject divalent ions, which can induce scaling [54]. In addition, NF can also reduce total dissolved salts (TDS) and remove microorganisms, turbidity and organic matter. 

### 3.7. Energy

Energy consumption, in kWh/m^3^ of product water, is a major parameter characterizing the performance of RO desalination plants [9,10,55]. Depending on seawater salinity, the energy consumption of a modern seawater RO system is 3.5–6 kWh/m^3^. The more water is recovered per unit of seawater the more energy is needed, but it can be theoretically reduced below 0.7 kWh/m^3^. Energy consumption comes from the various parts of the RO desalination plant, including feed-water intake, pretreatment, RO (high-pressure pumps and energy recovery devices), post-treatment and brine treatment/disposal. The largest energy consumption (usually between 60–80%), depending on feed water, local conditions, and technology employed, comes from the main section where RO takes place. Indeed, seawater RO desalination energy consumption is very high because of the low recovery ratio (25–40%) and the high operating pressure (60–70 bars). Therefore, maximum recovery of energy from the disposed brine is very important.

**Figure 6:**
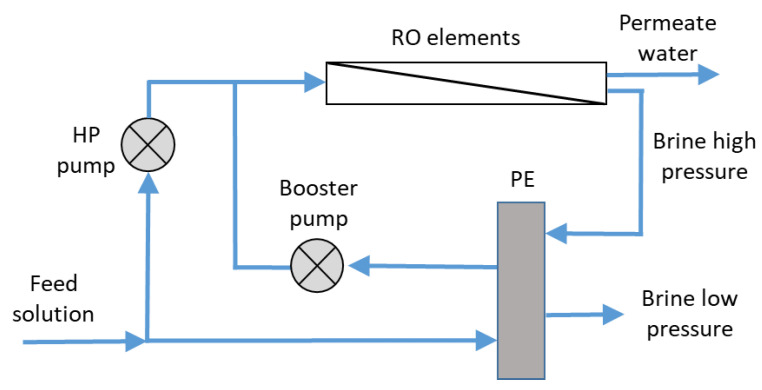
Pressure exchanger energy recovery system (adapted from [56]).

Different energy recovery systems are commercially available and have been implemented in several RO plants. For example, Avlonitis et al. [56] tested four different energy recovery systems (classical Pelton wheel, turbocharger, pressure exchanger and Pelton wheel commercialized by Grundfos company). These energy recovery systems have been applied in small and medium size RO plants. The most efficient recovery system was found to be a pressure exchanger by considering the recovery ratio and the specific energy divided by the recovery ratio (Figure 6). Much research is still ongoing to develop new energy recovery systems. For example, Song et al. [54] introduced a new piston-type integrated high pressure pump-energy recovery device that synchronously pressurizes the feed seawater and recovers the hydraulic energy from the concentrated brine. The utilization of this new device, instead of a single high-pressure pump, was shown to decrease both the payback period and the desalinated water cost.

The high energy required to run desalination plants remains a major drawback. Therefore, the idea of using renewable energies such as solar, wind, wave, geothermal and hydrostatic pressure, is attractive [15,16,17,18,57]. Their applicability strongly depends on the availability of renewable energy resources.

Solar energy has been extensively investigated as a potential source of energy for water desalination [58]. Solar energy may be used directly in solar stills or may be converted to electricity and then used in either thermal or membrane processes for desalination. Photovoltaic (PV) powered RO systems have been implemented in several places around the world, in countries such as Egypt, Jordan, and Australia.

Wind is an interesting source of energy as it is often available in coastal areas. Wind-powered RO plants have been implemented in several countries such as Croatia, Norway, and Australia. For example, a wind powered desalination plant was installed on the island of Gran Canaria [59]. The plant includes two wind turbines and a flywheel, which supply the energy to a complete desalination plant (eight RO modules, pretreatment and post-treatment facilities and control system). A similar plant has been run for more than 15 years by Solaires Canarias S.L. [60]. The cost of the plant was evaluated by assuming the combined use of RO and wind energy, a membrane life-time of 10 years, a total capital investment cost of 196,000 euros, and a profitability index of 1.3794. The internal rate of return was found to be as high as 225%, confirming the interest of such plants.

In addition, desalination plants can be supplemented by both wind and solar energies. These plants can be found in several countries, such as Sultanate of Oman, Israel, Mexico, and Germany. For example, the DESIRES^®^ (DESalting Island on Renewable Multi-Energy Supply) project proposed a floating island with a combination of several renewable energies such as wind, solar, and wave [61].

Wave energy is another possible energy source, especially in coastal regions and island nations. However, large investment costs render current wave-powered desalination technologies economically unrealistic. Recent research has focused on developing feasible wave-powered RO desalination techniques. For example, Brodersen et al. [62] studied the feasibility of direct-drive ocean wave-powered batch RO using a modelling approach. Seawater is used as the working fluid in a hydro-mechanical coupling and replaces the RO high-pressure pump with a hydraulic converter for direct-drive. System modelling shows that energy consumption and cost of water are competitive.

It is usually recognized that the optimization of energy requirement relies on conventional energy reduction possibilities, such as energy recovery systems, but also on several aspects including the development of ultra-high permeability membranes and fouling-resistant membranes, hybrid systems and renewable-energy-driven desalination [11]. Regulations to develop less energy-intensive desalination technologies should also help the implementation of these techniques [18].

### 3.8. Environmental Issues

The environmental impact of RO desalination is of major concern because of its extensive development over recent years. Several environmental issues have been reported, mainly attributed to the discharge of a brine of high salinity and high chemical concentration, but also to the influence of the intake on the marine environment, and to emission of air pollutants and greenhouse gases [7]. Several solutions are available to reduce environment problems. For example, the use of subsurface intakes for seawater RO desalination plants limits the use of chemicals in the pretreatment step and thus environmental issues [63]. Seawater is then filtered by natural sediments and rocks. The intake may be realized through wells of different geometries such as vertical, angle, and radial types or galleries. These structures can be built on the beach or in the seabed.

The reject water from the RO desalination plant has a salinity much higher than the seawater, and is called brine or brine-blowdown. The characteristics of the brine are related to the quality of the feed water, the desalination technology, the percent recovery, and the chemical additives used [64]. The brine may contain high levels of TDS, organic compounds and chemicals such as anti-scalants, antifoulants, and acids.

The brine is rejected directly into the sea, in evaporation ponds or injected into wells. Direct rejection into the sea influences microalgal, plant and animal life, and may also result in the formation of sludge. The degree of degradation depends on the total volume of the brine being released, its characteristics, the dilution rate prior to discharge, and the characteristics of the receiving water [65]. The effect of the brine on the environment also depends on the geometric installation of the discharge outfall. In open and well mixed water, adverse impacts are noticed mostly within 300 m from the discharge point. The effect is more pronounced in water located in shallow and/or semi-closed bays. Rejection in evaporation ponds is generally realized in inland RO desalination plants (in arid and semi-arid areas) where direct rejection into the sea is not possible and solar energy is abundant. Drawbacks are the space and surface needed, which affect the availability of soils. Dilution of the brine with seawater or water used can reduce environmental impact.

Another option to reduce environmental impacts associated with brine is the extraction of valuable compounds. Recovery of minerals from brine can be realized by precipitation, crystallization, adsorption, membrane distillation, or evaporation [8]. Hybrid techniques can also be implemented. For example, Ahmed et al. [64] described the extraction of dissolved minerals from brine by multiple evaporation and cooling. The compounds recovered include gypsum, sodium chloride, calcium carbonate, magnesium hydroxide, calcium chloride, and sodium sulphate used by various industries. Nevertheless, these processes are limited by several disadvantages such as high energy consumption, and requirement of large amounts of chemicals, and have limited applications at the industrial-scale level [8]. Improvements are needed to improve the separation of single elements from the others and to lower the overall cost of the processes to become more attractive.

### 3.9. Other Membrane Processes for Desalination

Forward osmosis (FO) and MD are more advanced alternatives membrane techniques compared to RO desalination [21,23]. They offer several advantages over RO, such as high salt rejection (MD), higher recovery of water (MD), and fewer pretreatment stages (MD, FO). They are being intensively investigated for implementation at large-scale and to improve their feasibility in terms of productivity, cost, and lower energy consumption to become competitive with RO.

#### 3.9.1. Forward Osmosis

**Figure 7:**
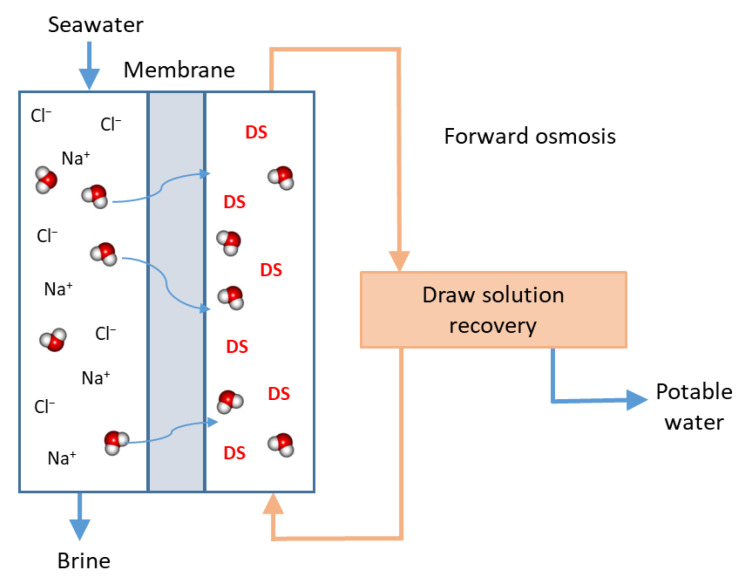
Schematic representation of a Forward Osmosis system.

FO uses the osmotic pressure difference between seawater and a highly concentrated draw solution (DS) [21,22,23]. The mass transfer is driven by the osmotic pressure gradient through a semi-permeable membrane. Water moves from the seawater across the membrane into the DS, while the salts and other dissolved solids are retained on their respective sides. Once the DS is diluted with fresh water, it is sent to the recovery process to be concentrated. The recovered water is then collected for distribution, while the regenerated DS is sent back to the FO plant (Figure 7). Appropriate selection of DS and its low cost recovery is important for feasible implementation. Ammonia-based solutions are usually used as the DS and can be recovered by moderate heating (60 °C). FO membranes are asymmetric with a thin active layer and a support layer. As in RO, concentration polarisation and fouling happen in FO. Two mechanisms are generally described: internal concentration polarisation inside the membrane pores, and external concentration polarisation that happens on both membrane surfaces.

Membranes used in FO are usually asymmetric thin-film composite RO membranes. However, the permeate water flux of these membranes is generally low due to internal concentration polarisation, especially in the dense thin layer. Several materials have been proposed to improve the performance of thin-film composite RO membranes, with the inclusion of nanomaterials such as zeolite, SiO_2_ and graphene oxide nanosheets. Nanoparticles can be incorporated into the support (mixed matrix membrane) or in the selective thin layer (thin-film nanocomposite) [19]. As in new developments in RO membranes, new membranes for FO are mostly developed at small scale and for short usage times.

Several developments are similar in FO and RO in seawater desalination. UF can be used as an effective pretreatment before FO, with addition of ferric chloride coagulation to improve filtration performance [66]. Similar strategies are also employed to reduce membrane fouling, such as pre-treatment, membrane surface modification and choice of appropriate operating conditions [20].

#### 3.9.2. Membrane Distillation

**Figure 8:**
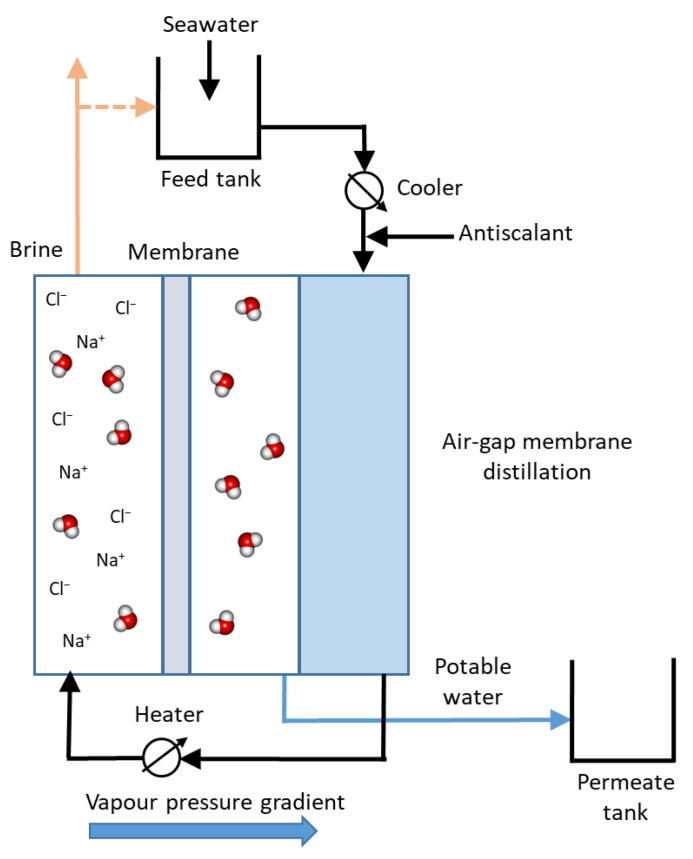
Schematic representation of an air-gap Membrane Distillation system.

MD is a membrane process that uses hydrophobic microporous membranes. The membrane separates a hot and cold stream of water, preventing flow of liquid water through the membrane and allowing flow of water vapour (Figure 8). The temperature difference produces a vapour pressure gradient that causes water vapour to pass through the membrane and condense on the colder surface. The water obtained has very high purity. MD can be conducted in different configurations that differ in the way the permeate is collected, the mass transfer mechanism through the membrane, and the driving force [67]. Various configurations of MD are possible, such as direct contact, air gap, sweeping gas and vacuum.

For desalination, the seawater obtained by cooling the condenser foil to, for example, 75 °C, creates a water vapour partial pressure difference between the two sides of the membrane and allows evaporation through the membrane. The water vapour condenses on the low-temperature side and distillate is formed. MD can be used as a substitute for desalination processes such as RO [68]. The advantages of MD are lower operating pressure and performance not limited by high osmotic pressure or concentration polarization.

The interest of using MD for desalination has been increasing worldwide, especially when coupled with solar energy. Several MD configurations have been tested and plants have been implemented. In the first example, two solar thermal MD units were developed and installed in Jordan [69]. Each unit consists of flat plate collectors, PV panels, spiral air gap MD module(s), and a data acquisition system. In the second example, the Memstill^®^ process was developed by TNO (Netherlands) for desalination of seawater by air gap MD carried out in a counter current flow configuration [70]. MD can also be used to reverse ED for the valorization of hypersaline waste brine to implement Zero Liquid Discharge and low-energy desalination (MD) [71].

#### 3.9.3. Electrodialysis

ED is an electric field driven membrane process. In an ED module, cation exchange and anion exchange membranes are alternatively stacked together, separated by flow spacers. ED is implemented for seawater and brackish water desalination in several plants; in particular, small and medium plants [72,73]. The cations migrate from the brackish water towards the negative electrode through the cation-exchange membranes which allow only cations to pass. On the other hand, the anions migrate towards the anode through the anion exchange membranes. Inverters are used to reverse the polarity of the electric field about every 20 min, to limit scaling. This process is called electrodialysis reversal (EDR). Solar and wind energy can supply ED in areas where sun and/or wind are highly available. For example, Veza et al. [74] built an ED/wind energy plant in Canaria Islands (Spain) for brackish water desalination.

In reverse electrodialysis (RED), a similar configuration to ED allows generation of electrical power from two salinity gradients [75]. The ion-exchange membranes are used for ion transport with the concentration difference as the driving force. ED and REV can be associated with desalination as a potential fresh water supply on small islands [76].

Despite its performance, seawater desalination by ED is generally considered to require too high an amount of energy to be competitive with RO. New ED configurations have thus been proposed such as an electrically segmented ED configuration and a hydraulically staged (i.e., multistage) ED configuration [77]. The multistage configuration had an average energy consumption of 3 kWh/m^3^ over an 18 days’ period, demonstrating the potential of multistage ED seawater desalination over RO.

## 4. Removal of Specific Compounds in Natural Water

### 4.1. Introduction

Seawater is a major source of drinking water. In several countries, another important source of drinking water is groundwater. Due to rock dissolution and/or industrial pollution, groundwater sources may contain several toxic molecules and particles such as synthetic organic chemicals such as pesticides, herbicides, industrial solvents and chemicals, inorganic pollutants such as arsenic, nitrate, and metals, and NOM and microorganisms such as protozoa, bacteria and viruses. These contaminants are traditionally removed by technologies such as adsorption, coagulation, flotation, ozonation, ion exchange, and pressure-driven membrane processes such as MF, UF, NF, RO, and membrane hybrid techniques. These techniques are often similar to those employed for seawater desalination and their advantages and limits are the same, such as ease of implementation in large plants, and fouling problems, respectively.

### 4.2. Natural Organic Matter

Natural waters contain colloids and NOM, i.e., molecules derived from the degradation of plants and microorganisms. NOM can be divided into dissolved organic matter (DOM) and particulate organic matter (POM). Humic substances are the major fraction of NOM and generally divided into three species: humic acid, fulvic acids and humin. NOM is a cause of colour in natural water sources and is not considered to be harmful to humans. However, NOM can generate disinfection by-products (DBPs) during chlorination of the RO system. DBPs are very toxic as they can generate cancers, and have to be removed before chlorination [78].

NOM removal is done by coagulation and flocculation followed by sand filtration or direct filtration [78]. Given more stringent water quality regulations, pressure driven membrane processes such as NF and RO are increasingly used; however, membrane fouling by NOM is a major limitation [79,80]. Multivalent cations, such as calcium and magnesium, react with NOM to form complexes, which results in a highly compacted fouling layer associated with rapid flux decline [79]. The rate of deposition onto the NF or RO membrane surface, and thus fouling, are also controlled by the coupling between the opposite forces of electrostatic repulsion and hydrodynamic force [79].

As in seawater desalination, pretreatment of fresh water sources can reduce membrane fouling and thus improve process efficiency [80]. For NOM removal, MF or UF can be associated with other processes such as adsorption on activated carbon, oxidation by iron oxide particles, photocatalysis, ozonation and electrocoagulation [81]. For example, pretreatment by coagulation immediately before the UF or MF membrane is effective in preventing membrane fouling and reducing the coagulant dose and the duration of water treatment compared to coagulation alone. However, the removal of NOM can be significantly affected by the type of coagulant, coagulation conditions, type of membrane, filtration conditions, and characteristics of the water to be treated [78]. Ozonation can also be used in combination with UF or MF to reduce membrane fouling by NOM. Catalysts, such as metal oxides, can be used simultaneously. For example, Park et al. [82] reported NOM removal using a hybrid process that combined ozonation with iron oxide nanoparticle-loaded membranes. Their results indicated that the reactive membrane-ozonation process enhanced NOM removal and reduced membrane fouling by generating hydroxyl radicals from the catalytic ozonation.

Renewable energies are also interesting alternatives to reduce costs associated with energy consumption in drinking water production systems. Systems based on UF driven by gravity have been developed and used at the small-scale, especially for decentralized production of drinking water in developing countries [83]. However, these systems are limited by low values of permeate flux (typically lower than 20 L m^−2^ h^−1^), and developments are needed to increase the permeate flux, while maintaining minimal need for maintenance.

### 4.3. Arsenic

In many places around the world, arsenic is present in drinking water due to natural geochemical phenomena or industrial pollution. Intensive consumption of arsenic contaminated water is the cause of various types of human diseases, including respiratory diseases, gastro-intestinal, liver and cardiovascular problems, and increasing risk of cancer. High arsenic concentrations are found in groundwaters at concentrations above the maximum contaminant concentration in countries such as China, Bangladesh and India. The maximum arsenic concentration stated by the WHO, US and European Union is 10 mg/L. In other countries such as India and China, this concentration may be higher (50 mg/L) [84,85].

Arsenic exists in groundwater in two predominant species: trivalent arsenite As(III) (H_3_AsO_3_) and pentavalent arsenate As(V) (H_3_AsO_4_) [84]. Both As(III) and As(V) are found in groundwater. Several processes can be used for arsenic removal, including oxidation, coagulation–precipitation, adsorption, ion exchange, and membrane separation [85]. Coagulation and adsorption provide good arsenic removal efficiency.

**Figure 9:**
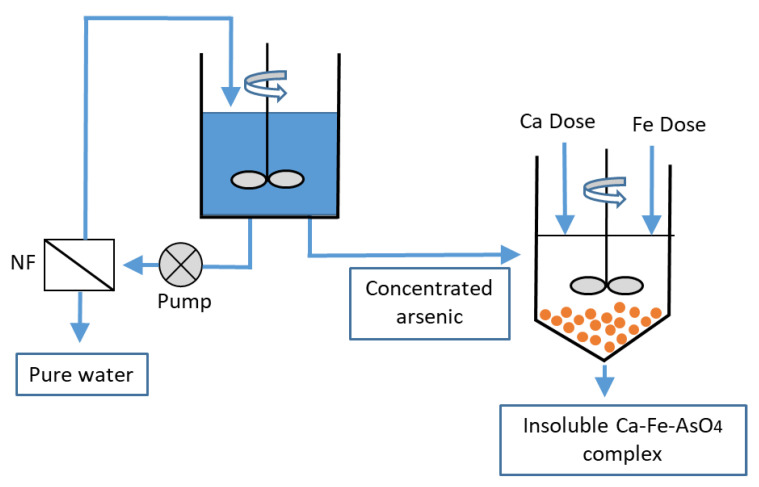
A nanofiltration–coagulation integrated system for separation and stabilization of arsenic from groundwater (adapted from [84]).

Membrane processes such as NF and RO have been largely used for arsenic removal. The greatest removal is obtained by RO, but RO has a high cost (plants and energy required). NF can remove arsenic in its As(III) form by steric effects, as it is uncharged, and As(V) by Donnan and steric effects, as it is negatively charged [84,86]. Therefore, water with a high As(III) concentration requires a pre-oxidation step by chemical oxidation (KMnO_4_, H_2_O_2_, OCl^−^ and S_2_O_8_^2−^), Fenton [Fe(II)/H_2_O_2_] or ozone (O_3_). Small particles (0.1–1 μm) are then obtained that are removed in a settling basin. For example, Pal et al. [84] removed trivalent and pentavalent arsenic by cross flow NF following a chemical pre-oxidation step for conversion of trivalent arsenic into a pentavalent form, with simultaneous stabilization of arsenic rejects for safe disposal (Figure 9). Ion exchange is another possible technique, its drawbacks being associated with resin regeneration and cost (resins and plant).

Hybrid membrane processes are often chosen to improve process efficiency (arsenic removal, less chemicals, and lower cost). For example, MF is an alternative to coagulation-flocculation-settling, where particles can be removed using a low-pressure pump without chemicals and the need for large space. The technique was successfully applied to the treatment of groundwater from a city in southern Colorado in the United States, and from Sonargan in Bangladesh [87]. Ozonation can also be used as a pretreatment. For example, Park and Choi [85] removed As(III)) using iron oxide nanoparticles-loaded membranes (to promote ozonation). Santoro et al. [88] implemented MD, photocatalysis and polyelectrolyte-enhanced UF for the treatment of arsenic contaminated water in Sila Massif (Italy), ensuring a complete removal of arsenic and a rational management of residual contaminants.

In several volcanically active regions, such as southern Peru, the presence of arsenic is associated with high boron concentrations [89]. Higher arsenic and boron removal is obtained by an increase in pH. Increasing the pH to 9.5 requires an additional cost evaluated between 0.03 and 0.08 $/m^3^.

### 4.4. Iron

Iron is often found at high concentrations is ground water, from 0.5 to 50 mg/L [25]. This is mainly due to dissolution of rocks and minerals. Iron is not toxic for humans but has several negative effects on drinking water, such as unpleasant taste and color; it can also induce the growth of ferro-bacteria. According to European regulations, the maximal total iron concentration in drinking water is 0.3 mg/L. Iron is mainly found in two states: Fe^2+^ and Fe^3+^.

Iron can be removed from ground water by several techniques, such as oxidation/precipitation/filtration, ion exchange, lime softening and membrane processes [25].

The oxidation/precipitation of soluble Fe^2+^ into insoluble iron hydroxides, especially ferric hydroxide Fe(OH)_3_, is done by adding chemicals (hypochlorite, permanganate) or dissolved gases (oxygen, chlorine, ozone) [90]. For water with a high iron concentration (>5 mg/L), aeration is the best way to oxidize the ferrous iron and avoid the use of chemicals. At lower iron concentration, ozonation or chlorination are usually chosen for oxidation. Iron hydroxide particles are then removed by sand filtration to obtain an iron concentration lower than 7 mg/L, and then by decantation.

Ferric and ferrous ions are not easily retained by NF, UF or MF, so, iron-based particles need to be formed before being filtrated. Iron-based complexes are obtained using chelating agents [25,91] or by oxidation (by biological treatment, sodium hypochlorite addition (prechlorination), potassium permanganate addition or air bubbling) to form ferric hydroxide particles. For example, iron and manganese were removed from groundwater by aeration, chlorine oxidation and MF at a water treatment plant in Taiwan Chen et al. [92].

Different process configurations are possible: (1) oxidation takes place in a reactor, and the ferric hydroxide suspension is then filtered by UF or MF, (2) the treatment is continuous, the water being added continuously to an aerated reactor and then the ferric hydroxide particles are eliminated by UF or MF [91]; (3) the reactor is coupled to UF or MF, the permeate and retentate being recycled in the reactor without continuous water addition. The second configuration is particularly attractive for groundwater treatment plants.

### 4.5. Nitrate

Nitrate is found at moderate concentrations in most groundwaters, but high concentrations are increasingly observed around the world, mainly resulting from intensive use of fertilizers. Nitrate is known to be harmful especially to infants and pregnant women [26]. This is due to the potential reduction of nitrate to nitrite ion which can bind with hemoglobin, thus diminishing the transfer of oxygen to the cells resulting in a bluish skin color often called “the blue baby syndrome”. This is a reason why the limit concentration in drinking water has been fixed by the WHO at 50 mg/L.

High nitrate concentrations limit the direct use of groundwater for human consumption in several parts of the world including Saudi Arabia, India and China [26]. For example, in the Nagpur district of Maharashtra in India, about 91% of the villages recorded use of groundwater with nitrate concentrations between 20 and 100 mg/L, and about 7% with concentrations higher than 100 mg/L. In Israel, nitrate concentrations higher than 70 mg/L have led to the closure of wells in the coastal aquifers, with an annual water loss of around 24 million m^3^ [93].

The techniques use for nitrate removal are ion exchange, ED and RO. Removal of nitrate by NF membranes is low due to the monovalency of nitrate. For example, Van der Bruggen et al. [94] found nitrate removal around 76% with a NF70 membrane (Dow/Filmtec). This concentration was said to be sufficient as a first step with ED or RO for complete removal. On the other hand, RO is very efficient for nitrate removal. RO has been implemented successfully in a rural area in South Africa [95]. Indeed, RO was found to remove 98% nitrate-nitrogen, from 42 mg/L to less than 1 mg/L. The RO brine was said to be suitable for stock watering if water recovery was kept low (approximately 50%) and if conditions for stock watering were met in terms of nitrate/nitrogen concentration, TDS and other constituent concentrations. Brine disposal for stock watering is a very convenient and cost-effective way of brine disposal in a rural area. However, the drawbacks of RO may be associated with its price, production of concentrated waste brines and their disposal [93].

Combination of NF and RO is a possible alternative. For example, Epsztein et al. [93] proposed a hybrid NF/RO process for nitrate removal due to the ability of NF to remove more chloride and sodium ions than nitrate ions. In a second stage, RO was applied to remove nitrate, and the RO permeate was mixed with the side stream of the NF stage to produce water with low nitrate concentration and a suitable composition with all required species and minerals. The hybrid process consisting of single and double NF stages followed by RO was able to reach water recoveries of 91.6% and 94.3%, respectively, based on Israeli regulations for drinking water and composition of brines discharged to the sewage.

### 4.6. Fluor

High fluoride concentrations are mainly found in water sources of countries in North and East Africa, and in India and China [27,28]. These high concentrations are due to natural dissolution of rocks and soils, and/or mining industries. At high concentrations, fluoride ions may be toxic for humans, with negative effects on teeth, bones or brain. Thus, the maximum concentration in drinking water recommended by the WHO is 1.5 mg/L.

Water defluoridation techniques include ion exchange, membrane processes, electrocoagulation, coagulation-precipitation and adsorption [27,28]. Ion exchange and membrane processes can decrease fluoride concentrations below recommended concentrations. Their drawbacks are their cost and the need to regenerate resins or membranes. Electrocoagulation is a very effective technique for fluoride removal but requires much energy, and the dissolution of the anode increases the aluminium concentration in water, which leads to a secondary pollution. The Nalgonda technique is a coagulation–precipitation technique with lime and aluminium as chemicals. This technique is largely implemented in India as it is very effective for fluoride removal. Drawbacks associated with this method include the formation of toxic soluble aluminium complexes, and an increase of pH and total dissolved solids, so an additional process is needed to eliminate chemicals. Adsorption is another technique, which has the advantages of being less expensive and simple to operate but has the disadvantage of being less effective.

NF is the best membrane process to remove fluoride from water as it very selective [27]. Indeed, the high hydration of fluoride ions increases their exclusion by organic NF membranes. For example, with the NF90 membrane, the fluoride concentration in the permeate increased with increasing fluoride concentration in the feed water, but fluoride was reduced to a satisfactory value for all concentrations (up to 1.5 mg/L) [96].

Following this theoretical study, Pontié et al. [97] proposed the first NF plant for defluoridation, which was built in Thiadiaye (Senegal) [97]. The skid-mounted system was a typical two-stage design plant typical for brackish water treatment, with a water tank of 1 m^3^ and a membrane area of 1338 m^2^ (36 spiral wound modules of the NF90-400 membrane). Cartridge filters (10 μm) were located upstream of the NF unit in order to limit particulates fouling. The F^-^ concentration in the feed water was 4.7 mg/L. With the NF90 membrane, the fluoride concentration decreased to a value of 0.6 mg/L.

### 4.7. Removal of Pesticides and Herbicides

Herbicides and insecticides are intensively used to control weeds (herbicides), insects (insecticides) and plant diseases that may affect the growth, harvest, and marketability of crops [29]. This results in their presence at very small concentrations (pg/L to ng/L) in surface and eventually in groundwaters. The potentially adverse health effects of herbicides and insecticides include increasing risk of cancer, genetic malformations, neuro-developmental disorders and damage to the immune system. Conventional methods such as particle coagulation–flocculation, sedimentation and dual media filtration, are ineffective for removing pesticide residues from water sources. Advanced treatments (such as oxidation by H_2_O_2_ or O_3_, and granular activated carbon /filtration) are effective but their limits are related to saturation of activated carbon, and toxic chemical by-products, which may develop in the GAC filters under some conditions.

In these last 20 years, NF and RO membranes were shown to remove of a large number of herbicides, insecticides and fungicides from various waters. Plakas and Karabelas [29] reviewed the NF and RO membranes employed together with reported pesticide rejection performance for 49 active substances. Factors affecting the removal of pesticides by NF and RO membranes include membrane characteristics (molecular weight cut-off, membrane material and charge), pesticides properties (molecular weight and size, hydrophobicity/hydrophilicity, polarity), and feed water composition (pH, solute concentration, ionic environment).

Zhang et al. [98] investigated the removal of two pesticides (atrazine and simazine) from different waters (distilled, tap and river water) using four types of NF membranes (DESAL 51 HL, DESAL 5 DL, UTC-20, UTC-60). The rejection of atrazine was found to be higher than the rejection of simazine; and the highest rejections were obtained with the UTC-20 membrane. The rejection of pesticides was higher in river and tap water than in distilled water, but the water flux was lower. This was explained by ion adsorption inside the NF membrane pores which modifies the rejection rates and water fluxes. In addition, the presence of NOM enhances the adsorption of pesticides onto the membranes surface and increases the size exclusion and electrostatic repulsion. In addition, these authors showed that pesticides were completely removed from water, with only a small fraction of salts using loose NF membranes (DESAL 51HL, N30F and NF270) in cascades. Rejection of pesticides (aldrin, atrazine, bentazone, dieldrin, and propazine) depended on specific properties of the solutes, such as molecular size and chemical structure (e.g., hydrophobicity).

### 4.8. Pharmaceuticals and Personal Care Products

Pharmaceuticals and personal care products (PPCPs) are well recognized trace contaminants of sewage, rivers, lakes, and groundwater [30,31]. PPCPs can have negative effects on humans and animals mainly because their residues enter and accumulate in food through contamination of water used in culture irrigation. Among pharmaceuticals, water can contain antibiotics, hormones, analgesics, anti-inflammatory drugs, blood lipid regulators, β- blockers, and cytostatic drugs and, as personal care products, preservatives, bactericides/disinfectants, insect repellents, fragrances, and sunscreen ultraviolet (UV) filters. In addition to PPCs, endocrine-disrupting chemicals (EDCs) are less specific and interfere with the functioning of natural hormones in animals such as fishes [99]. Some natural or synthetic compounds are considered to be EDCs, including pharmaceuticals, pesticides, industrial chemicals, phytoestrogens, and hormones excreted by animals and humans. PPCPs and EDCs are found in natural waters at a concentration below 1 μg/L.

Many processes have been proposed for reducing the concentration of PPCPs and EDCs in natural water [30,100]. Coagulation, flocculation, and precipitation processes are often ineffective for removing PPCPs and EDCs. Oxidative processes such as chlorination and ozonation can reduce the concentrations of several classes of contaminants; however, their efficacy depends on the contaminant structure and oxidant dose. Biological processes, such as activated sludge, biofiltration, and soil aquifer treatment can reduce the concentration of PPCPs and EDCs which are biodegradable and/or readily bind to particles. Activated carbon can remove nearly all PPCPs and EDCs; however, removal capacity is limited by contact time, competition with NOM and contaminant solubility.

Many studies have reported the treatment of natural or synthetic waters containing PPCPs and EDCs using RO and NF [99,100]. RO can efficiently remove almost all PPCPs, but its operational cost is relatively high since RO is operated under high pressure. NF membranes have shown high rejection for a wide range of PPCPs, but their performance is influenced by size exclusion and electrostatic and hydrophobic interactions, especially in the case of NF membranes with large pore size. Generally, larger molecules with a negative charge and higher hydrophilicity are more efficiently rejected. To improve the removal obtained by UF or MF, theses membrane processes can be associated with other techniques, such as activated carbon sorption and enzymatic degradation. For example, Snyder et al. [99] used granular activated carbon (GAC) and powdered activated carbon (PAC) as a pretreatment to membrane separation systems. Both GAC and PAC were effective for removal of PPCPs from water (more than 90% removal). However, the efficacy of GAC was influenced by NOM, which competes for binding sites. The authors also underline that the pressures required for RO and NF, as well as the thermal regeneration of GAC, require significant amounts of energy, which may lead indirectly to greater environmental impacts than the presence of trace contaminants.

## 5. Conclusions

This review presents classical and recent applications of membrane processes in the field of desalination and water treatment. Desalination is a classic and well-established technology with many plants built all around the world. Natural water treatment is also largely implemented in areas using natural water as water source. This review presents the main characteristics of these processes by focusing on membranes and devices, plants, membrane fouling, energy consumption, and environmental issues. Advances in desalination technologies could bring new solutions to natural water treatment. In desalination plants, renewable energy could be implemented to decrease energy consumption and environmental issues; environmental impact assessment requires further evaluation. Understanding membrane fouling in desalination could also provide valuable data for natural water treatments. Desalination and natural water treatment are thus closely linked, potentially offering new solutions in each field.

## Data Availability

Data is contained within the article.
